# The Strange Case of BCG and COVID-19: The Verdict Is Still up in the Air

**DOI:** 10.3390/vaccines8040612

**Published:** 2020-10-16

**Authors:** Radha Gopalaswamy, Natarajan Ganesan, Kalamani Velmurugan, Vivekanandhan Aravindhan, Selvakumar Subbian

**Affiliations:** 1 Department of Bacteriology, ICMR-National Institute for Research in Tuberculosis, Chennai 600031, India; radhagopalaswamy@gmail.com; 2 Queromatics, Phoenix, AZ 85339, USA; nat@queromatics.org; 3 Department of Zoology, Kongunadu Arts and Science College, Coimbatore 641029, India; kvelmurugan_zo@kongunaducollege.ac.in; 4 Department of Genetics, Dr. ALM PG IBMS, University of Madras, Taramani, Chennai 600005, India; cvaravindhan@gmail.com; 5 Public Health Research Institute at New Jersey Medical School, Rutgers University, Newark, NJ 07103, USA

**Keywords:** SARS-CoV-2, COVID-19, BCG, tuberculosis, pandemic, trained immunity

## Abstract

COVID-19, caused by a novel coronavirus, SARS-CoV-2, contributes significantly to the morbidity and mortality in humans worldwide. In the absence of specific vaccines or therapeutics available, COVID-19 cases are managed empirically with the passive immunity approach and repurposing of drugs used for other conditions. Recently, a concept that bacilli Calmette–Guerin (BCG) vaccination could confer protection against COVID-19 has emerged. The foundation for this widespread attention came from several recent articles, including the one by Miller et al. submitted to MedRxiv, a pre-print server. The authors of this article suggest that a correlation exists between countries with a prolonged national BCG vaccination program and the morbidity/mortality due to COVID-19. Further, clinical BCG vaccination trials are currently ongoing in the Netherlands, Australia, the UK, and Germany with the hope of reducing mortality due to COVID-19. Although BCG vaccination helps protect children against tuberculosis, experimental studies have shown that BCG can also elicit a non-specific immune response against viral and non-mycobacterial infections. Here, we summarize the pros and cons of BCG vaccination and critically analyze the evidence provided for the protective effect of BCG against COVID-19 and highlight the confounding factors in these studies.

## 1. Introduction

Currently, the world is witnessing an outbreak of COVID-19, caused by a novel coronavirus, SARS-CoV-2. The World Health Organization (WHO) declared COVID-19 as a global pandemic on March 11, 2020. With its ability to infect through the respiratory tract and transmit through aerosol, SARS-CoV-2 causes significant morbidity and mortality in humans worldwide. At present, there are no vaccine or therapeutic interventions for COVID-19 [1,2,3].

With several ongoing clinical trials to test potential vaccines for COVID-19 at different phases [4], a lot of debate has gone into using bacilli Calmette–Guerin (BCG) vaccine to reduce COVID-19 morbidity. The BCG vaccine is primarily used in tuberculosis (TB)-endemic countries to protect children against TB [5]. Apart from TB, BCG is useful against several other infectious and non-infectious diseases by eliciting a heterologous host response and trained immunity [6,7,8,9,10,11,12,13,14].

Several recently published articles have argued that BCG vaccination can reduce the morbidity due to COVID-19 [15,16,17]. Remarkably, a pre-print report by Miller et al. indicates that a correlation exists between countries with a prolonged national BCG vaccination program and the morbidity/mortality due to COVID-19 [17]. Since a nationwide vaccination program can be beneficial to public health, it is logical to investigate the possible association between vaccination-induced immune responses and the morbidity/mortality of an ongoing pandemic, such as COVID-19. Concomitantly, clinical BCG vaccination trials have been started in a few countries, including the Netherlands, Australia, the UK, and Germany. These trials are primarily aimed at evaluating the protective efficacy of the BCG vaccine against SARS-CoV-2 infection in healthcare workers and/or the elderly population [18]. In contrast, the World Health Organization (WHO) released a note on 12 April 2020, stating that there is no evidence that BCG vaccination protects people against COVID-19 [19].

It is our perspective that the current scientific evidence for the usefulness of a BCG vaccination in controlling COVID-19 death is insufficient and warrants additional preclinical and clinical data. Recent reports suggest that BCG vaccination negatively correlates with the death rate among COVID-cases [17,20,21]. These studies are mostly statistical analyses of country-wise data on BCG vaccinations and deaths due to COVID-19. Although these reports are useful to gain some insight into the correlation between BCG vaccination and COVID-19 deaths, they also have limitations. For example, a study by Miller et al. has taken a correlational approach, based on a simple and specific study design with a multivariable analysis. The authors attempted to positively correlate COVID-19 mortality to the long-standing national BCG vaccination policies adopted in lower- and middle-income countries, compared to the higher income group countries, such as the USA, Italy, Lebanon, the Netherlands, and Belgium, which do not have a national BCG immunization policy. The study assesses the number of deaths (per million inhabitants) due to COVID-19 and reports that the middle- and high-income group countries with BCG vaccination policies had much lower mortality than the same group without a BCG vaccination. Further, the study presents a positive correlation between the start time of the mandated BCG vaccination policy and the reduced mortality rate due to COVID-19. The study eventually observes that adopting a robust and national-level BCG vaccination policy slows down the spread of COVID-19 in the middle- and high-income countries as compared to the countries in the same group with no universal BCG vaccination policy [17]. A similar approach and conclusion have also been reported by Klinger et.al [21]. Together, these studies allude to the possible beneficial effects of adopting a prolonged, nationwide BCG vaccination policy as a new protective measure to control the mortality of the current COVID-19 pandemic. However, as the authors of these studies acknowledged, there are several confounding factors that should be taken into consideration (see Discussion in Section 4 below) before concluding that the BCG vaccination indeed can significantly reduce the number of deaths due to COVID-19. In the present article, we summarize the immune response elicited by BCG and highlight some critical issues that are important in evaluating the potential benefit of the BCG vaccination in preventing mortality among COVID-19 cases.

## 2. BCG and Immune Response to Infection: A Mixed Bag

BCG is a live attenuated strain of *Mycobacterium bovis,* a member of the pathogenic *Mycobacterium tuberculosis* (Mtb) complex organisms. At present, BCG is the only vaccine approved by the WHO to prevent TB, a deadly contagious bacterial disease of humans. The original BCG vaccine was developed by Albert Calmette and Camille Guerin at the Pasteur Institute of Lille, France, between 1908 and 1921 after several hundred passages, and has been in use since 1921 [22].

Initially, BCG was given to infants by the oral route, and later on changed to a subcutaneous route [22]. Since the development of the original strain, 49 substrains of BCG have been produced and used worldwide [23]. BCG is the most widely administered vaccine, with about 90% coverage of the world population [24]; over 120 million people receive BCG each year. This vaccine shows a varied success rate (0% to 80%) in preventing TB in children [25,26,27,28,29]. Although BCG is protective against severe forms of TB in infants and children, the protective immunity generated by BCG wanes after 10–15 years of vaccination [30]. The South Indian BCG trial has shown that BCG vaccination failed to offer any significant protection against the adult form of pulmonary TB [31]. However, randomized and case-control trials have shown a consistently high protective efficacy for BCG (60% to 80%) against severe forms of TB, such as miliary TB and TB meningitis in children [32,33]. Further, the absence of prior exposure to any mycobacteria can increase BCG’s efficacy against pulmonary and, to a certain extent, miliary and meningeal TB [30]. This pre-exposure either masks or blocks the effect of BCG and considered an important reason for the variable efficacy of BCG [34]. Thus, in TB-endemic countries, BCG is given to 90% of neonates and infants at birth, except for those with symptomatic HIV infections, as part of the national childhood immunization program [24]. A BCG atlas database is available to check the universal BCG vaccination policy status worldwide [5,35].

## 3. A Case for the Non-Specific Immune Response of BCG for Protection against COVID-19

In addition to eliciting an antigen-specific immune response against TB, BCG has also been reported to exhibit non-specific, immunomodulatory effects against diverse diseases, including nematode, fungal, and viral infections, bladder cancer, allergy, and asthma [6,7,11,14]. There are two primary mechanisms postulated to explain BCG’s non-specific effects: heterologous immunity and trained immunity. In heterologous immunity, it is proposed that the vaccine antigens elicit a cross-reactive host response and antibody production against other pathogens. Several vaccines, including the BCG, have been shown to produce a heterologous protective immunity, leading to an improved response against non-mycobacterial pathogens. In trained immunity, the innate immune cells, such as macrophages, can be trained to develop a proinflammatory response upon stimulation with BCG, which can also help protect the host against subsequent infection by non-mycobacterial, fungal or viral pathogens. However, the contribution of such a non-specific immune response elicited by BCG, in the setting of COVID-19, is not fully understood.

## 4. Implied Effects of BCG on the Heterologous Immune Response

In vitro and in vivo studies using fungal, viral, and bacterial infectious agents have shown that the innate immune cells, such as monocytes, dendritic cells, and NK cells, can be trained, such that these cells can non-specifically protect against infection [11]. BCG, Lipopolysaccharide (LPS), and Glucan, as well as other biomolecules derived from fungi and bacteria, have been shown to train the innate immune cells [13]. This T- and B-cell-independent immunity conferred by BCG is believed to be characterized by metabolic alterations, the upregulation of innate immune receptors, such as TLRs (toll-like receptors), and cytokine responses, which are primarily controlled by epigenetic modifications of the stimulated host cells [6,11,12,14,36]. Most of the data for the BCG-induced trained immunity were generated using short-term (up to a week) in vitro stimulation assays with primary cells or cell lines that showed an increased production of proinflammatory cytokines, including TNF-α and IL-1β [8,9,10]. The BCG-induced trained immune response in human blood-derived macrophages was noted for up to three months but no longer than a year [9].

There have been various epidemiological, clinical, and immunological studies that reiterate BCG’s effects in protecting against viral infections. Studies using animal models of DNA and RNA viruses, including herpes simplex, influenza A, vaccinia, and Japanese encephalitis infection, have shown enhanced protection by BCG vaccination [37]. However, the challenge of animal models of BCG vaccination and viral infections is that they do not reflect the complexities in human populations and cannot be considered equivalent.

Epidemiological and clinical studies suggest that vaccination with some strains of BCG can reduce the viral loads in individuals inoculated with attenuated Yellow fever virus (YFV) or the Influenza H1N1 virus, which indicates a possible role for heterologous and/or trained immunity [37,38]. However, the YFV study was conducted in healthy volunteers of 25–35 years of age, who received the BCG vaccine for travel to TB-endemic countries. This study design is strikingly different from conventional neonatal BCG vaccination programs adapted in TB-endemic countries. Further, the heterologous immune response of BCG inferred from in vitro experiments using LPS-stimulated PBMCs, and in vivo studies using the severe combined immunodeficient (SCID) mice are inadequate to explain the complexity observed in a population of neonatal BCG-vaccinated individuals. Thus, more robust longitudinal cohort studies are needed to evaluate both BCG-induced heterologous protection and the mechanism of trained immunity in humans.

Further, in a population with varied age groups, the effect of BCG-induced immunity would wane disproportionally and offer a pool of subjects who would respond differently to a non-mycobacterial infection. Additionally, the key cytokine implicated in BCG’s cross-protection, IL-1β, is neither specific to trained immunity nor is sufficient to elicit a robust protective immune response against a range of pathogens. The in vitro assays used in these studies are preliminary and only indicate an immune response under the tested conditions [39,40]. Interestingly, previous research using antisera and T-cells from immunized mice have suggested that none of the childhood vaccines, including BCG, oral polio vaccine (OPV), hepatitis B vaccine (HBV), measles, mumps, rubella and varicella vaccine (MMRV), and others, have shown any significant cross-protection against SARS-CoV infection, which caused an epidemic in 2003 [40].

Although arguments made based on a statistical analysis of epidemiological data for the correlation of BCG and COVID-19 at different stages of this pandemic are interesting, and may have implications for controlling the COVID pandemic, there are some confounding points concerning the correlation between BCG vaccination and its protective immune response against COVID-19, as listed below:

(1) Metrics for quantifying the BCG vaccination policies: The parameters for defining national BCG vaccination policies are based on assumptions. A lack of any standard, quantitative metric mentioned to evaluate the successful enforcement of the BCG vaccination policy and using income levels as a surrogate indicator to reflect the success of the vaccination policy, sets a weak premise for the correlation analysis. Additional socio-economic parameters such as the overall educational qualification of the study population and job definitions should be considered while studying the confounding effect of socio-economic differences. Further, the metrics for quantifying BCG vaccination is confounded by a misclassification bias and/or measurement errors (see below). Thus, multiple, quantitative metrics, including income, nature of the healthcare system, health-seeking behavior in the community, cultural dogma and social setting, should be considered when evaluating the success of universal BCG vaccination policies [39]. Further, there is variation in BCG vaccination coverage among countries of different incomes. For example, lower-middle income group countries, such as India, have an 88% coverage, while low-income countries have a 78% coverage [41]. Belgium, one of the high-income group countries included in the Miller et al. study, has used BCG for 67 years, until 1989, then vaccination was targeted for a specific group of people, such as migrants. Since 2013, the BCG vaccination is considered optional in Belgium. A similar situation has also been reported for Italy, another high-income country. Therefore, the current lack of implementation of a universal BCG vaccination should not be construed as no BCG vaccination at all during any period within the average life expectancy period of a country (e.g., Belgium and Italy). In these countries, an analysis of the COVID-19 death rate among vaccinated and non-vaccinated individuals would provide a clear picture on the beneficial effect of BCG vaccination. In addition, the correctional factors added for statistical analysis of epidemiological data introduces a potential bias. For example, upper-middle and high-income group countries were combined as a single group for analysis, while low-income group countries were ignored in the report by Miller et al. [17]. Similarly, the Klinger et al. study stratified the age of study population into: below 24 years, 25–64 and above 65 years [21] to analyze the impact of the BCG vaccination on the 4-month (29 January to 21 May 2020) death rate of COVID-19. There was no rationale given for the assumptions made in these studies for such classification of data. Further, in most of the TB-endemic countries, a BCG vaccination is given at birth, and the vaccine-induced immunity wanes with time up to a decade. Even in trained immunity studies, the protective effect was shown only for about a year. Thus, there is a disconnect between the biological factors associated with neonatal national BCG vaccination programs and the artificially introduced values to adjust for “factors” in the statistical analysis, which could skew the interpretation of the real impact of BCG vaccination in reducing the mortality due to COVID-19.
(2) Supporting correlational data: There have been insufficient details presented on the epidemiological studies regarding confounding factors that correlate with the income metrics of nations to their BCG vaccination policies. Without this, the direct correlation (BCG vaccine policy vs. COVID-19 cases/deaths) assumes little significance. Similarly, there is a disparity in average life expectancy between low-, middle- and high-income group countries. For example, Italy’s (an upper-income country) average life expectancy is 84.01 years, whereas it is 70.42 for India, a country with a low-middle income. This disparity in life expectancy is a crucial confounding factor. Since the risk of death from COVID-19 increases with age beyond 65, the life expectancy is an important factor, but was not considered in the statistical analysis of the epidemiological studies.
(3) Outlier analysis: The explanation for China and Japan as outliers is incomplete and insufficient. The disbanding and weakening of the policy during China’s Cultural Revolution (a subjective point) make the age of the subjects’ fatalities to be roughly 50s or younger. However, China’s data [42] on COVID-19 fatalities (one of the earliest reports to become public and widely cited) have shown increased mortality in the elderly population, primarily in their 70s; the highest death rate was seen among individuals of >80 years. These data have now been confirmed through other studies. Similarly, Japan has seen a spike in the number of COVID-19 cases and gone into a National Emergency [43].
(4) False sense of security from BCG-induced trained immune response: The role of the BCG vaccine in training innate immune responses has long been debated and is still fraught with caveats [15,44]. For the correlation to be translated into causation, the mechanisms of BCG vaccine-induced trained immunity should be consistent with the findings in human clinical studies. Since this is yet to be definitively settled, it is presumptive to hint at the success of a national BCG vaccination policy, which pertains mostly to neonates, in controlling the morbidity and mortality due to COVID-19 in mostly adults. Further, this would deliver a false sense of security among the BCG-vaccinated population with COVID-19 [22,44].
(5) BCG strain variation and protective effect: It remains unclear if the protective effect of BCG is targeting the SARS-CoV-2 per se or the secondary health effects caused by the virus, including sepsis or inflammation. Further, the strain variation in BCG across different countries would have elicited different levels of the protective immune response to a wide range of pathogens in various populations. Similarly, exposure to environmental microbes, particularly non-tuberculous mycobacteria (NTM), is another major factor that shapes the host immune response. However, the impact of such response in modulating the heterologous protection conferred by BCG is not fully understood. Finally, the host immune response contributed by differential environmental exposure, such as air particulate matter and toxic pollutants in various countries, on SARS-CoV-2 infection remains unknown. Understanding these critical confounding factors is vital to delineate the contribution of BCG-induced immune protection against COVID-19.
(6) Effect of immigrant population with childhood BCG vaccination: Some high-income countries, such as the USA, have a significant immigrant population who would have received a childhood BCG vaccination in their country of birth [45,46]. The proportion of such a population would affect the disease transmission and mortality of COVID-19. Further genetic diversity arising due to ethnic differences can have a major impact on interpreting these data. However, these aspects have not been addressed in any of the epidemiological studies that attempted to associate BCG vaccination with reduced COVID-19 deaths.
(7) Data coverage: Other confounding factors, such as the accuracy and reliability of data on the number of COVID-19 cases and deaths, and variability in the nature and coverage of COVID-19 testing procedures across different countries are not adequately discussed in the correlation studies. Age, sex or vaccination for other infections, such as polio, mumps, rubella, etc., are also confounding factors, as discussed in a recent article [35]. In fact, in another study, the authors found a lack of correlation between countries giving BCG vaccinations and mortality due to COVID, when the testing rates were included in the statistical analysis performed independently in their study [47]. Consistent with this study, Hamiel et al. reported that the outcome of COVID-19 in adults with or without a prior BCG vaccination within a single country (Israel) was not statistically significantly different [20]. Thus, epidemiological and ecological studies that create a fallacy due to possible inference of observation from in vitro experiments to individuals with heterogeneous immune responses and extending the study group averages to the total population should be interpreted with great caution.
(8) Additional parameters—Though the epidemiological studies recognize the influences of other parameters contributing to the disparities in fatalities across nations—e.g., standard of care—there are additional factors that are significant enough to skew the analysis. For example, there is a disparity in the definition of “Deaths due to COVID-19” and the reporting system across different countries. The incidence of co-morbid conditions, such as HIV infection, diabetes, and hypertension can impact the death rate due to COVID-19. However, the current International Form of Medical Certificate of Cause of Death, as prescribed by the WHO, does not discriminate deaths due exclusively to SARS-CoV-2 infection or due to complications from co-existing morbidities in COVID-19 cases [48]. Thus, there is a possibility for variation between the number of reported deaths and the actual number of deaths due to COVID-9 among different countries.
(9) The stage of the spread has changed widely for different nations [35,49] and it has been fluctuating. Further, the authors have not specified the names of the nations in other income categories, including key countries, such as India, to evaluate the claims more thoroughly. For example, as of 2 October 2020, the cumulative cases in the USA (high-income group without a national BCG vaccination program) was 7,160,476 and the cumulative death rate due to COVID-19 was 205,666 (2.872%), while these values for India (low-middle-income group with national BCG vaccination program) were 6,394,068 and 99,773 (1.560%) [50]. However, the first reporting of cases/deaths due to COVID-19 occurred much earlier in the USA, compared to India, which has the second most COVID-19 deaths as of 2 October 2010. The trend of the mortality rate would change in the coming days/weeks/months. Therefore, it is very premature to conclude that BCG protects against COVID-19 deaths in countries with a national BCG vaccination policy. Infrastructure readiness—in response to the inundation of the healthcare system by a sudden surge in cases and hospitalizations, including the number of beds and ventilators available and accessible. Stockpiling of resources—access to healthcare resources, including the availability and access to COVID-19 test kits, the sensitivity and specificity of such diagnostic kits, personal protective gears, as part of pandemic preparedness, are unclear. The knowledge of public—the health-seeking behavior among the men and women of different countries varies strikingly, which is closely related to the social, cultural, and literacy rate, but this factor was not accounted for in the epidemiological studies [17,21].

## 5. Conclusions

The ability of BCG to confer protection against COVID-19 is an exciting concept that requires convincing evidence from extensive preclinical and clinical studies. Our perspective has also been supported by recently published articles [16,35,47] that suggest the need for more scientific evidence for the protective efficacy of BCG against COVID-19. Although it is tempting to proclaim that BCG can prevent COVID-19 deaths, there are several confounding factors, and circumstantial evidence exists in studies, such as that of Miller et al. [17], which precludes any valid presumptions on the causal link between BCG vaccination and protection against COVID-19. Several other groups have extensively debated the role of BCG in defense against SARS-CoV-2 infection. Most of them converge at the point that the ecological and epidemiological data would be insufficient and clinical trials are pertinent. The correlation of BCG immunization data with COVID-19 cases and deaths is primarily biased by vaccination policy, economy, age, sex, ethnicity, calculation of mortality data, availability of the healthcare system, pandemic awareness, testing rates, proper reporting of cases, including their mortality and reiterated by many groups worldwide. In future studies, it would be interesting to see the effect of BCG on SARS-CoV2 infection and COVID-19 through proper clinical cohort studies [2,3,16,17,18,20,21,35,36,39,47].
